# Prolonged Medical Cannabis Treatment is Associated With Quality of Life Improvement and Reduction of Analgesic Medication Consumption in Chronic Pain Patients

**DOI:** 10.3389/fphar.2021.613805

**Published:** 2021-05-19

**Authors:** Joshua Aviram, Gil M. Lewitus, Yelena Vysotski, Ben Yellin, Paula Berman, Anna Shapira, David Meiri

**Affiliations:** Faculty of Biology, Technion-Israel Institute of Technology, Haifa, Israel

**Keywords:** medical cannabis, chronic pain, phytocannabinoids, terpenoids, quality of life

## Abstract

**Introduction:** Chronic non-cancer pain (CNCP) is one of the most prevalent indications for medical cannabis (MC) treatment globally. In this study, we investigated CNCP parameters in patients during prolonged MC treatment, and assessed the interrelation between CNCP parameters and the chemical composition of MC chemovar used.

**Methods:** A cross-sectional questionnaire-based study was performed in one-month intervals for the duration of six months. Subjects were adult patients licensed for MC treatment who also reported a diagnosis of CNCP by a physician. Data included self-reported questionnaires. MC treatment features included administration route, cultivator, cultivar name and monthly dose. Comparison statistics were used to evaluate differences between the abovementioned parameters and the monthly MC chemovar doses at each time point.

**Results:** 429, 150, 98, 71, 77 and 82 patients reported fully on their MC treatment regimens at six one-month intervals, respectively. Although pain intensities did not change during the study period, analgesic medication consumption rates decreased from 46 to 28% (*p* < 0.005) and good Quality of Life (QoL) rates increased from 49 to 62% (*p* < 0.05). These changes overlapped with increase in rates of (-)-Δ^9^-*trans*-tetrahydrocannabinol (THC) and *α*-pinene high dose consumption.

**Conclusion:** Even though we observed that pain intensities did not improve during the study, QoL did improve and the rate of analgesic medication consumption decreased alongside with increasing rates of high dose THC and *α*-pinene consumption. Understanding MC treatment composition may shed light on its long-term effects.

## Introduction

Chronic non-cancer pain (CNCP) is defined as pain that is not resolved in an expected time-frame, does not respond to acceptable analgesic treatment, and lasts more than three months (Tunks et al., 2008). CNCP has a negative impact on sleep (Vaughan et al., 2018) and on patients’ physical, psychological and social wellbeing, which when combined, are often defined as health-related quality of life (QoL) (Jensen et al., 2007). QoL is one of the important outcome domains being measured in the evaluation of pain treatment effectiveness and has been suggested to be indicative of treatment success (Börsbo et al., 2009).

CNCP treatment is based on combinations of pharmaceutical analgesics (non-steroidal anti-inflammatory drugs (NSAIDs), anticonvulsants, antidepressants, but mostly by opioids (Chou et al., 2009) and complementary non-pharmacotherapy treatment (e.g. physical therapy, dry needling, behavioral therapy) (Cherkin et al., 2014; Lee et al., 2014). Nevertheless, as the 'opioid crisis' continues (Volkow and Collins, 2017), newer, safer options for the treatment of CNCP merit investigation.

In recent years, CNCP has become a commonly approved indication for treatment of medical cannabis (MC) (Fitzcharles and Eisenberg, 2018). As such, CNCP is the most researched indication for MC treatment, with over 40 randomized controlled trials (RCTs), which produced many reviews, meta-analyses and even systematic reviews of systematic reviews (Stockings et al., 2018). However, the low to moderate level of evidence, and the often inconsistent findings of RCTs (Pratt et al., 2018), left the issue of cannabinoid treatment for CNCP unresolved. In addition, most RCTs have analyzed the effectiveness and safety of a particular component of MC (i.e., (-)-Δ^9^-*trans*-tetrahydrocannabinol, THC), or in some cases, a combination of THC and cannabidiol (CBD) in a fixed ratio (Aviram and Samuelly Leichtag, 2017). However, patients rarely consume these cannabis-based medications (CBMs), but rather whole-plant cannabis products, which contain many other biologically active constituents, including additional phytocannabinoids (Berman et al., 2018) and terpenoids (Shapira et al., 2019). Hence, full spectrum analysis of MC is required to enable a broader understanding of its clinical potential and safety.

Therefore, this prospective, cross-sectional study examined over an extended period of time pain-related parameters such as pain intensities, sleep timing, QoL and analgesic medication consumption, as reported by CNCP patients under MC treatment; and moreover, it investigated the associations between the abovementioned parameters and the MC chemovar (phytocannabinoids and terpenoids) doses patients consumed.

## Materials and Methods

### Study Population

#### Inclusion Criteria

Patients were eligible to participate in this study if they were Hebrew speaking, aged ≥18 years, were diagnosed for CNCP by a physician and had a standing MC license for the treatment of CNCP. Israeli Ministry of Health (IMOH) regulations of MC state that a physician may request a license for a patient only if that patient meets specific indications, such as CNCP, and only following the exhaustion of all traditional pharmaceutical medications (e.g., opioids, NSAIDs, anticonvulsants and antidepressants) for a duration of at least one year. Contraindications for MC license approval were primarily pregnancy, lactation, diagnosis of schizophrenia and insufficient exhaustion of approved analgesics.

### Exclusion Criteria

Patients were not eligible to participate in this study if they did not have an authorized MC license or if their MC license was terminated by their physician during the study period.

### Medical Cannabis in Israel

At the time the data was collected, physicians in Israel decided in collaboration with the patient on one of two approved (by the IMOH) routes of administration, either inflorescences for smoking and vaporization, and/or MC extracts dissolved in vegetable oil for sublingual use. The physician-determined monthly dose of MC generally started at 20 g, as indicated by the IMOH, with dose increases subject to MCU approval (Landshaft et al., 2019). Physicians provided consultation for selection of a specific or combination of MC cultivars and patients made the final decision on the MC cultivar/s. Every patient went through a personal trial-and-error process to find the cultivar or the combination of cultivars that best met his/her needs.

### Instruments

#### Online Survey

Data collection was carried out online by the secured survey technology Qualtrics^®^ (Provo, Utah, version 12018) (Qualtrics, 2015).

### Study Questionnaires

At each time point, demographic information included age, gender, BMI, illicit use of MC prior to obtaining a license, tobacco and alcohol consumption habits and physical activity status. Data on pain characteristics included the least, average and worst weekly pain intensities (on a numerical pain scale (NPS) ranging from 0-10) and pain etiology. Specific information on pharmaceutical analgesics were also reported. Two validated questionnaires were utilized in this study: 1) the quality of life questionnaire, EuroQol (EQ5) (Brooks, 1996), that was validated to Hebrew by Horowitz et al. (2010), (Horowitz et al., 2010). The EQ-5 questionnaire is comprised of five individual questions, relating to the five main domains of quality of life (mobility, self-care, daily activities, pain/discomfort and anxiety/depression), the patients are asked to rate their current state, from 1 (no problem) to 3 (severe problem). The final questionnaire score is then summed, ranging from 5-15.2) the sleep timing section of the Pittsburgh sleep quality index (PSQI) (Smyth, 1999) that was validated to Hebrew by Shochat et al. (2007), (Shochat et al., 2007) included questions on time to bed (e.g., 21:00), sleep latency (minutes), waking time (e.g., 07:00) and sleep duration (hours). Additionally, patients reported on their MC treatment characteristics, including administration route, cultivator brand, cultivar name, total monthly dose (g) and the monthly dose of every cultivar (g). Patients also reported on adverse effects (AEs) that they attribute directly to the MC treatment.

### Phytocannabinoid and Terpenoid Profiling of Cannabis Chemovars

Air-dried medical cannabis chemovars were obtained from several Israeli medical cannabis cultivators. Reagents, analytical standards and general methodologies for phytocannabinoid and terpenoids extraction and analysis from *Cannabis* were performed according to previously published methods (Berman et al., 2018; Shapira et al., 2019).

Briefly, for phytocannabinoid extraction, 100 mg of ground cannabis flowers were accurately weighed and extracted with 1 ml ethanol. Samples were agitated in an orbital shaker at 25°C for 15°min, and then centrifuged at 4,200 rpm. A fraction of the supernatant was collected and filtered through a 0.22 µm PTFE syringe filter and diluted in the ratios of 1:9, 1:99 and 1:999 v/v cannabis extract to ethanol. Phytocannabinoid analyses were performed using a Thermo Scientific ultra-high-performance liquid chromatography (UHPLC) system coupled with a Q Exactive™ Focus Hybrid Quadrupole Orbitrap mass spectrometer (MS, Thermo Scientific, Bremen, Germany). The chromatographic conditions were according to Baram et al. (2019), (Baram et al., 2019). Identification and absolute quantification of phytocannabinoids was performed by external calibrations (Berman et al., 2018).

For terpenoid analysis, 10 mg of ground cannabis flowers were weighed in 20 ml amber HS rounded bottom vial and immediately sealed with a magnetic 32 mm PTFE septa cap. Terpenoids were separated using a Trace 1,310 gas chromatograph (Thermo scientific, Germany) coupled to a TSQ 8000 Evo triple quadrupole mass spectrometer (Thermo scientific, Germany), equipped with a DB-35MS UI capillary column (30°m × 0.25°mm × 0.25°μm, Agilent, US). A Pal RTC autosampler (CTC Analytics, Switzerland) for automated static headspace injections (SHS) was used, 1 ml of a sample’s gas phase, prepared after 30 min agitation of a flower sample with 140°C temperature, was injected in GC injection port with a split ratio 1:50. Identification and absolute quantification of terpenoids was performed in MS/MS mode by external calibrations as described by Shapira et al. (2019), (Shapira et al., 2019).

In order to reduce the variability between analyzed cultivars, only phytocannabinoids and terpenoids with minimum average concentrations of 0.1 g and 400 ppm, respectively, were reported.

### Study Procedure

The study was approved prior to data collection by the institutional ethics committee of the Technion (# 011-2016). The data for this prospective cross-sectional study was gathered between 2017 and 2019. Participants were selected from an existing database of Israeli patients with a pre-existing MC license for various indications (i.e., not naïve to cannabis). This database has been operational from April 2016 To January 2021. The database was a nationwide project managed by Prof. David Meiri's team at the Technion and was not linked to any particular cultivator, nor to a specific medical unit. Patients agreed electronically to disclose their email address for future studies, and that also reported having a diagnosis of CNCP. These patients received an email with an explanation of the study design and a link to the online questionnaire. The same questionnaire was sent automatically every month for six months (i.e., a total of six follow-up questionnaires were produced, T_1_-T_6_), unless patients requested to be removed from the contact list; hence, no information on reasons for study withdrawal were collected. No financial compensation was offered to participating patients. This was an observational study with no intervention components, thus, registration on the Clinical Trials Register was not required. The phytocannabinoids and terpenoids of clinically administered cultivars from most approved cultivators in Israel were analyzed routinely by LC-MS and SHS-GC/MS/MS, respectively**.** It is important to note that the chemical analyses were performed on the inflorescence cultivars received from the cultivators and not directly from the patients, then matched to the patients according to the record of the cultivar(s). Specific phytocannabinoid and terpenoid monthly doses of each patient were calculated. The STROBE statement checklist for cohort studies is presented in the Supplementary materials (Methods S1).

### Statistical Analysis

R software (V.1.1.463) with the lme4 (Bates et al., 2014)*,* tidyverse (Wickham, 2019)*,* atable (Ströbel and Haynes, 2019), sjPlot (Lüdecke, 2019), pheatmap (Kolde, 2015), and arsenal (Heinzen et al., 2019) packages were used to analyze differences between time points in outcome measures by the Pearson's Chi-squared test for categorical measures and the Kolmogorov-Smirnov test for numeric measures. For the effect of size and confidence interval (CI) we utilized a Cohen's *d* test. Dichotomized parameters (i.e., monthly doses of cannabinoids and terpenoids) were chosen a cut point of their median distribution at T_1_. QoL score was divided to better and worse according to none-moderate (5–8) and severe-extreme (9–15), respectively. The MC cultivars were clustered based on their cannabinoids and terpenoids concentrations for descriptive purposes; Ward's method of agglomerative hierarchical clustering was selected, as it produced the most homogenous clusters and is commonly used in medical studies. Clustering was based on Euclidean distance between z-score normalized data. To assess associations between changes in outcome measures and MC chemovar dose consumption, we used generalized logistic mixed-effect regression models. Generalized logistic regression models were utilized in order to assess interactions. A Shapiro-Wilk test of normality demonstrated a non-normal distribution for all parameters; thus, data are presented as median and inter quartile range (quartiles 25 and 75, IQR). Differences were considered significant at the *p* < 0.05 level. Incidences are presented as number and percentage of patients. Due to the exploratory nature of the study, no sample size limits were calculated prior to data collection and as such, no corrections for multiple comparisons have been made. Since most study parameters did not change significantly between time points, only their T_1_ demographic characteristics are presented in the text, unless noted otherwise.

### Role of the Funding Source

The study was sponsored by the Evelyn Gruss Lipper Charitable Foundation. The study sponsors had no role or influence on the study or on this submission.

## Results

### Sample

From a contact database of 3,218 patients with prior MC license for various indications, 1,550 patients (48%) reported having a diagnosis of CNCP. Among these CNCP patients, 688 (44%), 266 (39%), 166 (24%), 135 (20%), 138 (20%) and 152 (22%) patients responded to our invitation to participate in the study at T_1_, T_2_, T_3_, T_4_, T_5_ and T_6_, respectively. Among them, we selected only those that consumed MC only by inflorescence inhalation and not by other administration routes and reported fully on their MC treatment regimen including the MC administration route, cultivator brand, cultivar name, total monthly dose (g) and the monthly dose of every cultivar (g). These patients consisted of 429 out of 688 (62%) at T_1_, 150 out of 266 (56%) at T_2_, 98 out of 166 (59%) at T_3_, 71 out of 135 (53%) at T_4_, 77 out of 138 (56%) at T_5_ and 82 out of 152 (54%) at T_6._]. These patients represent the sample that is reported and analyzed in this paper. Importantly, all patients (*n* = 429) reported at T_1_. From these patients, 223 (52%) reported on at least one follow-up, 118 (28%) reported on at least two follow-ups, 68 (16%) reported on at least three follow-ups, 43 (10%) reported on at least four follow-ups and 26 (6%) reported on five follow-ups. Notably, the sample demographics did not change between T_1_-T_6_, and consisted mainly of males (*n* = 275, 64%), with a median age of 42 (35–52) (Table 1).

**TABLE 1:** Demographic characteristics of the study sample at one month intervals within a six-month period.

Measure	T_1_ (N = 429)	T_2_ (N = 150)	T_3_ (N = 98)	T_4_ (N = 71)	T_5_ (N = 77)	T_6_ (N = 82)	Statistic (*p*)
	Number of patients (%)						
Gender							
Male	275 (64)	99 (66)	63 (64)	44 (62)	50 (65)	52 (63)	0.40^A^ (1.0)
Female	154 (36)	51 (34)	35 (36)	27 (38)	27 (35)	30 (37)	
Median (IQR)							
Age (years)	42 (35–52)	40 (33–53)	41 (32–54)	39 (34–55)	40 (32–53)	42 (35–53)	1.47^B^ (0.92)
BMI	24 (21–27)	24 (22–27)	24 (21–28)	25 (22–28)	25 (22–27)	24 (21–27)	3.84^A^ (0.57)
Number of patients (%)							
Tobacco smoking consumption							
Yes	222 (52)	77 (52)	56 (57)	39 (55)	36 (47)	38 (47)	5.71^B^ (0.84)
Alcohol consumption							
Yes	155 (36)	52 (34)	41 (42)	26 (36)	27 (34)	26 (31)	4.50^B^ (0.92)
Physically active							
Yes	164 (38)	50 (33)	33 (34)	26 (37)	32 (42)	29 (35)	2.38^B^ (0.79)

IQR, inter quartile range; BMI, body mass index; A, Kolmogorov-Smirnov test; B, Pearson's Chi-squared test; T_1_-T_6_, one to six months follow ups, respectively.

### Pain Parameters Characteristics

**FIGURE 1:**
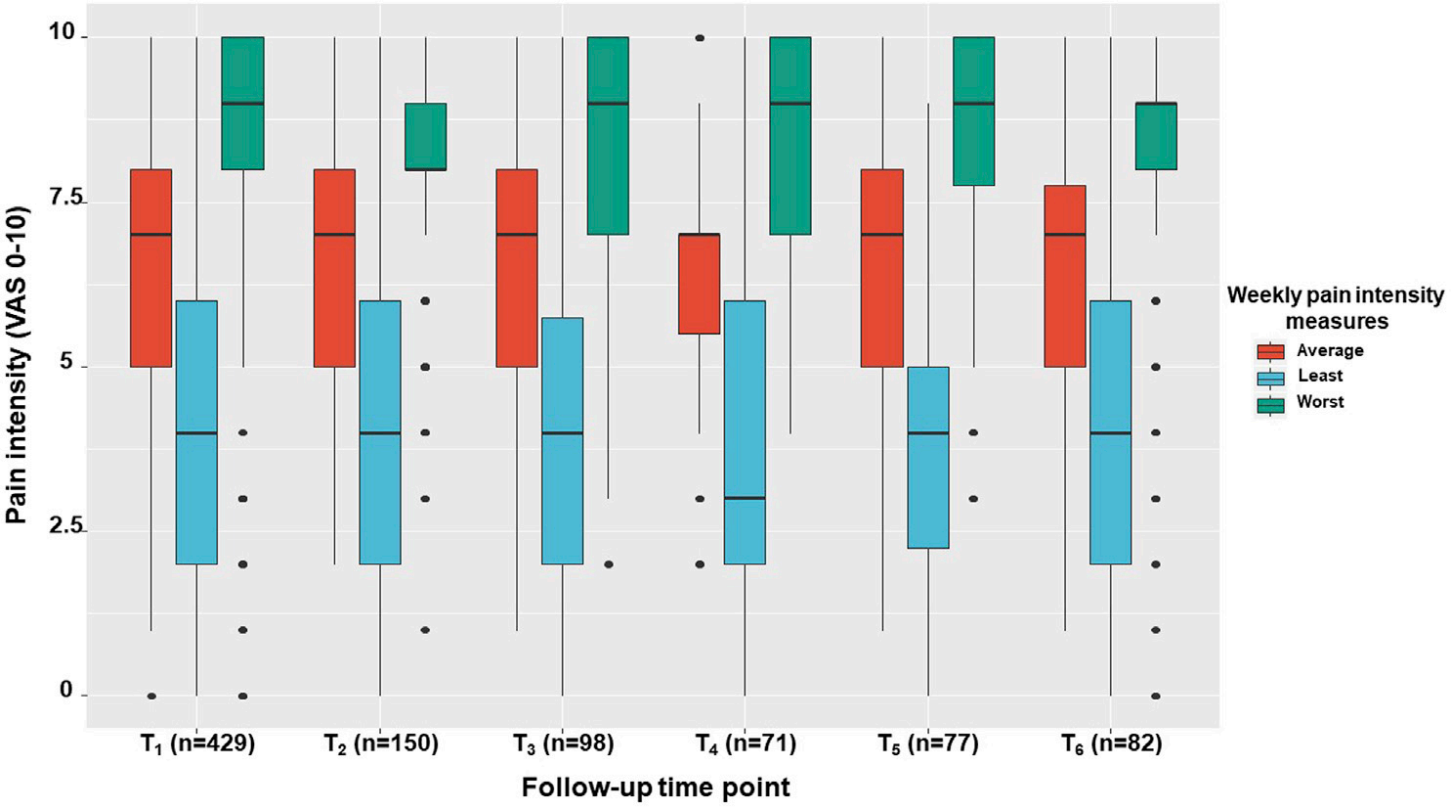
Pain intensity measures across study period. The median, inter quartile range, total range and outliers for the average, least and worst pain intensities are demonstrated for the study sample at one month intervals within a six-month period. VAS, Visual analogue scale; T_1_, T_1_-T_6_, one to six months follow ups, respectively.

Pain etiologies did not vary between follow-up time points. Many concomitant pain etiologies were reported, so we present data on the frequency of having each pain etiology component, either as a single pain diagnosis or in combination with other diagnoses. Hence, in descending order, pain etiology components consisted of neuropathic pain (*n* = 312, 73%), musculoskeletal pain (*n* = 256, 60%), headaches and nociplastic pain (*n* = 111 and *n* = 113; 26% for both), and visceral pain (*n* = 62, 16%) (Table 2). Notably, the least, average and worst weekly pain intensities did not vary significantly between follow up points (χ^2^_(5)_ = 0.23, 3.66 and 1.61; *p* > 0.05, respectively). Specifically, the least pain intensity was 4 (2–6), the average pain intensity was 7 (5–8), and the worst pain intensity was 9 (8–10) (Figure 1).

**TABLE 2:** Pain etiology characteristics of the study sample at one month intervals within a six-month period.

Measure	T_1_ (N = 429)	T2 (N = 150)	T_3_ (N = 98)	T_4_ (N = 71)	T5 (N = 77)	T_6_ (N = 82)	Statistic (*p*)
Number of patients (%)							
Pain etiology component#							
Neuropathic	312 (73)	107 (71)	70 (71)	46 (65)	46 (60)	57 (70)	6.52^B^ (0.26)
Musculoskeletal	256 (60)	82 (55)	56 (57)	39 (55)	41 (53)	42 (51)	4.75^B^ (0.66)
Headaches	111 (26)	36 (24)	23 (23)	19 (27)	16 (21)	22 (18)	4.05^B^ (0.91)
Nociplastic	113 (26)	38 (25)	26 (27)	18 (25)	18 (23)	23 (28)	2.94^B^ (0.99)
Visceral	62 (14)	20 (13)	11 (11)	11 (15)	6 (8)	11 (13)	4.97^B^ (0.67)

IQR, Inter quartile range; T_1_-T_6_, 1–6 months follow ups, respectively; #, pain etiologies exceed 100% due to cumulative diagnoses (i.e., one or more); A, Kolmogorov-Smirnov test; B, Pearson's Chi-squared test.

**FIGURE 2:**
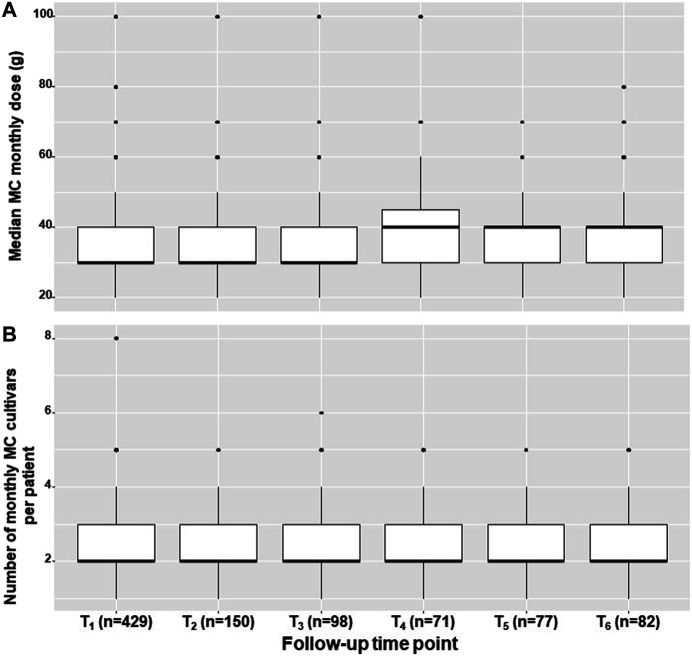
MC treatment measures across study period. The median, inter quartile range, total range and outliers for the monthly medical cannabis dose **(A)** and number of medical cannabis cultivars that are combined in a month period **(B)**, displayed for the study sample at one month intervals within a six-month period. MC, Medical cannabis; g, grams; T_1_-T_6_, one to six months follow ups, respectively.

In addition, there was no change in the monthly MC dose (g) of 30 (30–40) χ^2^_(5)_ = 7.92, *p* = 0.16) and in the number of monthly combinations of MC cultivars (2-3 per patient, χ^2^_(5)_ = 7.78, *p* = 0.17) over the study period (Figure 2). Additionally, no change in pain frequency (e.g., constant, few times per day, once-twice per day, few times per week, less than three-four times per month and less than one time per month) was observed (χ^2^_(5)_ = 21.06, *p* = 0.89). Notably, most patients (*n* = 227, 53% at T_1_) reported on constant pain frequency.

### CNCP Associated Measures and Analgesic Medications Consumption

Along with pain intensity measures, we evaluated two more parameters traditionally associated with CNCP, QoL and sleep timing. We found no significant changes during the study period in sleep latency, sleep duration and time in bed. Nonetheless, following division of the QoL questionnaire score (0–15) to better (0–8) and worse (9–15) subgroups, we observed an increase in the rates of patients' 'better' QoL scores from 49% (*n* = 210) at T_1_, to 62% (*n* = 51) at T_6_ (Table 3).

**TABLE 3:** Sleep timing, quality of life and analgesics consumption of the study sample at one month intervals within a six-month period.

Measure	T_1_ (N = 429)	T_2_ (N = 150)	T_3_ (N = 98)	T_4_ (N = 71)	T_5_ (N = 77)	T_6_ (N = 82)	Statistic (*p*)
Median (IQR)							
Sleep latency (min)							
	30 (15–60)	30 (15–48)	30 (15–60)	25 (10–60)	20 (10–40)	25 (10–45)	4.6^A^ (0.45)
Sleep duration (h)							
	6 (4.7–6.8)	6 (5–7)	6 (5–7)	6 (5–7)	6 (5–7)	6 (5.1–7)	4.4^A^ (0.48)
Time in bed (h)							
	7 (6–8)	7.1 (6–8)	7.5 (6–8.2)	7 (6–8)	7.2 (6.5–8)	7.2 (6–8)	1.0^A^ (0.96)
Number of patients (%)							
Quality of life (EQ-5, 0–15 score)							
Better (5–8)	210 (49)	78 (52)	56 (57)	45 (63)	46 (60)	51 (62)	10.9^B^ (0.05)
Worse (9–15)	219 (51)	71 (47)	42 (43)	26 (37)	30 (39)	31 (38)	
Analgesics consumption							
Yes	198 (46)	70 (47)	33 (34)	22 (31)	31 (40)	23 (28)	17.2^B^ (0.005)

IQR, Inter quartile range; min, minutes; h, hours; T_1_-T_6_, one to six months follow ups, respectively; A, Kolmogorov-Smirnov test; B, Pearson's Chi-squared test.

Additionally, we found that during the study period there were significant descending rates of individuals that reported consuming analgesic medications, from 46% (*n* = 198) at T_1_, to 28% (*n* = 23) at T_6_ (χ^2^_(5)_ = 17.24, *p* < 0.005). Among the individuals that consumed analgesic medications, no changes were observed in consumption rates of specific analgesic types during the study period, including over the counter analgesics, with 13% (*n* = 54) at T_1_ (χ^2^_(5)_ = 4.05, *p* = 0.54), NSAIDs with 8% (*n* = 36) at T_1_ (χ^2^_(5)_ = 3.6, *p* = 0.59), weak opioids with 14% (n = 61) at T_1_ (χ^2^_(5)_ = 4·7, *p* = 0·45), strong opioids with 19% (*n* = 83) at T_1_ (χ^2^_(5)_ = 6.9, *p* = 0.22), anticonvulsants with 10% (*n* = 43) at T_1_ (χ^2^_(5)_ = 2.1, *p* = 0.54) and antidepressants with 16% (*n* = 70) at T_1_ (χ^2^_(5)_ = 2.1, *p* = 0.71). Notably, there was a trend of reduction in daily morphine equivalent dose consumption, from a median of 0 (0–2.3) mg at T_1_, to 0 (0–0) mg at T_6_ (χ^2^_(5)_ = 10.45, *p* = 0.06). Specifically, we observed a decrease in daily mean ± SD consumption of morphine equivalent dose from 21 ± 91 mg at T_1_, to 5.2 ± 27.0 mg at T_6._

### MC Treatment Safety

Overall, MC-related AEs (i.e., at least one AE report) were reported by the majority (*n* = 368, 86%) of the patients at T_1_. However, most of these AE rates did not vary significantly during the study period (χ^2^_(5)_ = 1.4, *p* = 0.92). In descending order of frequency, AE reports at T_1_ which did not change significantly during the study included central nervous system (*n* = 259, 60%), psychological (*n* = 191, 45%), visual (*n* = 146, 34%), musculoskeletal (*n* = 132, 31%) and cardiovascular (*n* = 42, 10%) AEs. Notably, gastrointestinal AEs were reported at significantly increasing rates during the study, from 70% (*n* = 301) at T_1_ to 83% (*n* = 68) at T_6_ (χ^2^_(5)_ = 12.2, *p* < 0.05). The most frequent specific AE were fatigue, dry mouth and thirst, with 46% (*n* = 196), 38% (*n* = 163) and 31% (*n* = 135) rates at T_1_, respectively (Table 4).

**TABLE 4:** Specific medical cannabis related adverse effects rates of the study sample at one month intervals within a six-month period.

	Follow-up time points					
	T_1_ (n = 429)	T_2_ (n = 150)	T_3_ (n = 98)	T_4_ (n = 71)	T_5_ (n = 77)	T_6_ (n = 82)
	No. of patients (%)					
Central nervous system						
Confusion	45 (10)	16 (11)	11 (11)	11 (15)	9 (12)	8 (10)
Disorientation	10 (2)	6 (4)	5 (5)	1 (1)	2 (3)	2 (2)
Impaired attention	60 (14)	31 (21)	20 (20)	6 (9)	7 (9)	12 (15)
Dizziness	38 (9)	13 (9)	14 (14)	6 (9)	8 (10)	11 (13)
Increased awareness	60 (14)	21 (14)	13 (13)	12 (17)	11 (14)	13 (16)
Decreased awareness	29 (7)	13 (9)	3 (3)	4 (6)	5 (7)	6 (7)
Decreased physical sensation	17 (4)	12 (8)	5 (5)	1 (1)	2 (3)	4 (5)
Intoxication feeling	13 (3)	40 (5)	30 (5)	31 (5)	30 (6)	30 (6)
Impaired balance	37 (9)	14 (9)	10 (10)	6 (9)	4 (5)	9 (11)
Fatigue	196 (46)	76 (51)	51 (52)	31 (44)	39 (51)	36 (44)
Impaired memory	79 (18)	36 (24)	20 (20)	14 (20)	18 (23)	17 (21)
Impaired coordination	19 (4)	7 (5)	7 (7)	3 (4)	1 (1)	6 (7)
Impaired speech	23 (5)	9 (6)	1 (1)	4 (6)	1 (1)	4 (5)
Gastrointestinal						
Abdominal discomfort	64 (15)	22 (15)	16 (16)	13 (18)	12 (16)	17 (21)
Abdominal pain	55 (13)	20 (13)	14 (14)	14 (20)	11 (14)	17 (21)
Heartburn	30 (7)	15 (10)	9 (9)	9 (13)	7 (9)	8 (10)
Nausea	54 (13)	19 (13)	14 (14)	8 (11)	14 (18)	18 (22)
Vomiting	15 (4)	5 (3)	4 (4)	1 (1)	2 (3)	1 (1)
Diarrhea	31 (7)	16 (11)	8 (8)	6 (9)	7 (9)	11 (13)
Decreased appetite	64 (15)	26 (17)	12 (12)	9 (13)	10 (13)	14 (17)
Increased appetite	102 (24)	33 (22)	19 (19)	17 (24)	15 (19)	16 (20)
Sweet cravings	97 (23)	33 (22)	21 (21)	19 (27)	16 (21)	23 (28)
Bad taste	46 (11)	15 (10)	9 (9)	9 (13)	4 (5)	7 (9)
Thirst	135 (31)	52 (35)	34 (35)	26 (37)	21 (27)	31 (38)
Dry mouth	163 (38)	67 (45)	35 (36)	32 (45)	31 (40)	43 (52)
Psychological						
Unusual thinking	23 (5)	5 (3)	5 (5)	3 (4)	3 (4)	4 (5)
Anxiety	41 (10)	16 (11)	15 (15)	6 (9)	5 (7)	11 (13)
Dysphoria	84 (20)	24 (16)	18 (18)	11 (15)	11 (14)	15 (18)
Hyperactivity	18 (4)	7 (5)	1 (1)	1 (1)	0	3 (4)
Euphoria	18 (4)	3 (2)	3 (3)	4 (6)	4 (5)	7 (9)
Loss of time sensation	27 (6)	10 (7)	6 (6)	1 (1)	1 (1)	8 (10)
Forgetfulness	69 (16)	27 (18)	12 (12)	11 (15)	14 (18)	19 (23)
Nervousness	65 (15)	25 (17)	12 (12)	5 (7)	11 (14)	19 (23)
Stress	71 (17)	28 (19)	18 (18)	15 (21)	14 (18)	12 (15)
Tantrums	29 (7)	13 (9)	7 (7)	5 (7)	5 (7)	11 (13)
Musculoskeletal						
Joint pain	76 (18)	28 (19)	20 (20)	16 (23)	13 (17)	16 (20)
Limb weakness	58 (14)	19 (13)	15 (15)	6 (9)	6 (8)	9 (11)
Tremor	25 (6)	9 (6)	7 (7)	4 (6)	7 (9)	6 (7)
Spasms	41 (10)	14 (9)	15 (15)	4 (6)	5 (7)	8 (10)
Cardiovascular						
Palpitations	42 (10)	7 (5)	6 (6)	2 (3)	4 (5)	6 (7)
Visual						
Blurred vision	33 (8)	15 (20)	9 (9)	5 (7)	4 (5)	11 (13)
Red eyes	79 (18)	39 (26)	26 (27)	12 (17)	18 (23)	21 (26)
Dry eyes	69 (16)	33 (22)	19 (19)	14 (20)	16 (21)	22 (27)
Miscellaneous						
Headaches	3 (1)	0	0	0	0	0
Tinnitus	0	1 (<1)	0	0	0	0
Energy decrease	0	1 (<1)	0	0	0	0
Flu like symptoms	0	0	0	0	0	1 (1)
Hypoglycemia	1 (<1)	0	0	0	0	0

T_1_-T_6_, one to six months follow ups, respectively; AEs list comprised based on the report by Aviram et al., (Aviram and Samuelly Leichtag 2017); Miscellaneous AEs were reported as text as explanation to "other AEs".

### MC Treatment Complexity

**FIGURE 3:**
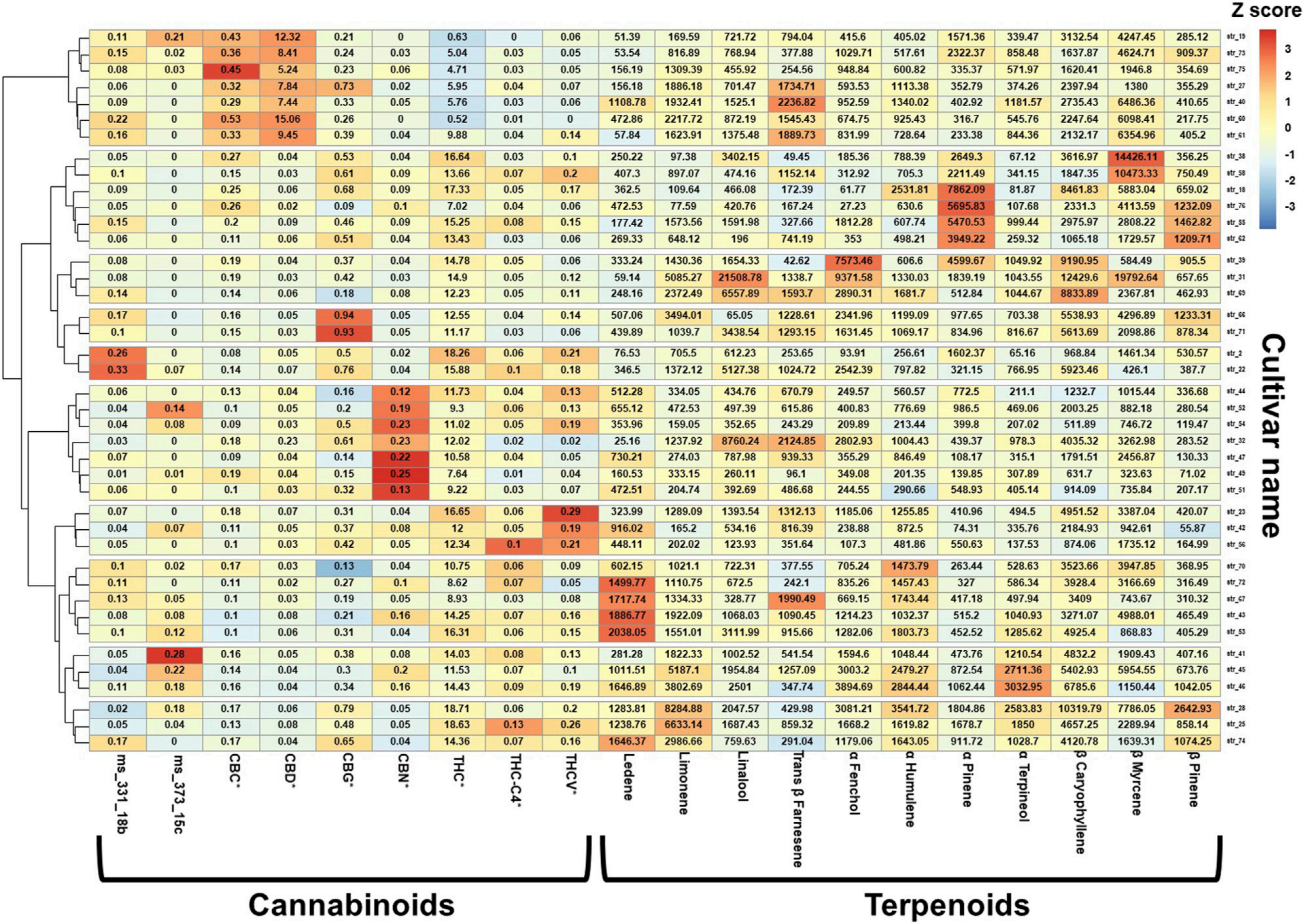
Cannabinoids and terpenoids relative dose in the consumed cultivars. The values in each box are the absolute concentrations of the specific phytocannabinoid (% w/w) or terpenoid (ppm) within each cultivar, and the color scale represents the z-scaled phytocannabinoid and terpenoid concentration variations between cultivars. *For each phytocannabinoid, the concentrations of the acid and its neutral counterpart were summed and reported as the total content; Method used: package "pheatmap", function pheatmap, with the (default): distance measure used in clustering rows "euclidean", clustering method used is "complete" on z scored data scaled by row; THC (-)-Δ9-trans-tetrahydrocannabinol; CBD, cannabidiol; CBC, cannabichromene; CBG, cannabigerol; CBN, cannabinol; THC-C4 (-)-Δ9-trans-tetrahydrocannabinol-C4; THCV (-)-Δ9-trans-tetrahydrocannabivarin.

The complexity of MC treatment is due in part to the variety of available cultivars, distributed by several authorized cultivators, the option given to patients to consume more than one cultivar, and varying doses of cultivars consumed in the same month. Consequently, 350 unique MC cultivar combinations were reported in the current study by 429 patients, consisting of 41 unique cultivars. Figure 3 shows a *z*-score heatmap of the major phytocannabinoid and terpenoid concentrations in the 41 cultivars, arranged according to hierarchical clustering of phytocannabinoid and terpenoid groups. The 41 cultivars were clustered into 10 groups according to chemical composition. Notably, 34 (83%) of the cultivars were THC-dominant and only 7 (17%) cultivars were CBD-dominant.

### MC Treatment Characteristics

As noted in the methods section, we evaluated inflorescence consumption only after decarboxylation, meaning via smoking or vaporization and not sublingual consumption of oil extract, because it is less valid to compare the effect of a specific MC chemovar dose between two different pharmacokinetic routes. Thus, patients that reported sublingual consumption of oil extract(s) (*n* = 125 at T_1_) were not included in the analysis. Consequently, at T_1_, 324 (76%), 86 (20%) and 19 (4%) patients reported on exclusive MC consumption via smoking, vaporization or a combination of these two administration routes, respectively. Notably, this variable remained stable during the study period (χ^2^_(5)_ = 5.04, *p* = 0.89). The median treatment duration prior to this study was 2 (1-4 IQR) years ranging from patients treated for over one year to 13 years.

Following division of all measured MC chemovars to low and high monthly dose consumption based on their distribution of consumption in our sample, we found that most phytocannabinoid and terpenoid doses consumption did not vary across the study period. However, we observed a trend for increased rate of consumption of chemovars with high THC from *n* = 198 (46%) at T_1_ to n = 47 (57%) at T_6_ (χ^2^_(5)_ = 9.94, *p* = 0.07) and a significant increase for rate of consumption of chemovars with high *α*-pinene from n = 125 (29%) at T_1_ to 133 (40%) at T_6_ (χ^2^_(5)_ = 11.85, *p* < 0.05) (Table 5).

**TABLE 5:** Low/high monthly doses rates of the predominant phytocannabinoids and terpenoids consumed by patients of the study sample at one month intervals within a six-month period.

	T_1_ (N = 429)	T_2_ (N = 150)	T_3_ (N = 98)	T_4_ (N = 71)	T_5_ (N = 77)	T_6_ (N = 82)	Statistic (*p*)
Number of patients (%)							
Chemovar monthly dose (mg) - pPhytocannabinoids							
∆^9^-*trans*-tetrahydrocannabinol (THC)							
Low (123–4,892)	231 (54)	81 (54)	49 (50)	28 (39)	33 (43)	35 (43)	9.9 (0.07)
High (4,892–14,123)	198 (46)	69 (46)	49 (50)	43 (61)	44 (57)	47 (57)	
THC-^4^ **C**							
Low (0.21–20)	228 (53)	80 (53)	42 (43)	27 (38)	39 (51)	38 (46)	8.8 (0.11)
High (20–69)	201 (47)	70 (47)	56 (57)	44 (62)	38 (49)	44 (54)	
Tetrahydrocannabivarin (THCV)							
Low (0.15–50)	232 (54)	76 (51)	46 (47)	28 (39)	35 (45)	37 (45)	7.8 (0.17)
High (50–378)	197 (46)	74 (49)	52 (53)	43 (61)	42 (55)	45 (55)	
Cannabidiol (CBD)							
Low (3–20)	223 (52)	81 (54)	48 (49)	29 (41)	40 (52)	33 (40)	7.2 (0.20)
High (20–4,576)	206 (48)	69 (46)	50 (51)	42 (59)	37 (48)	49 (60)	
Cannabigerol (CBG)							
Low (16–143)	222 (52)	81 (54)	49 (50)	27 (38)	34 (44)	41 (50)	6.6 (0.25)
High (143–619)	207 (48)	69 (46)	49 (50)	44 (62)	43 (56)	41 (50)	
Cannabinol (CBN)							
Low (0.21–19)	223 (52)	77 (51)	48 (49)	35 (49)	38 (49)	35 (43)	2.5 (0.76)
High (19–106)	206 (48)	73 (49)	50 (51)	36 (51)	39 (51)	47 (57)	
Cannabichromene (CBC)							
Low (10–55)	223 (52)	77 (51)	52 (53)	33 (46)	37 (48)	32 (39)	5.5 (0.35)
High (55–356)	206 (48)	73 (49)	46 (47)	38 (54)	40 (52)	50 (61)	
331–-18b							
Low (4–29)	216 (50)	83 (55)	53 (54)	38 (54)	36 (47)	41 (50)	2.2 (0.81)
High (29–157)	213 (50)	67 (45)	45 (46)	33 (46)	41 (53)	41 (50)	
373–-15c							
Low (0–20)	233 (54)	69 (46)	45 (46)	32 (45)	40 (52)	36 (44)	6.8 (0.23)
High (20–113)	196 (46)	81 (54)	53 (54)	39 (55)	37 (48)	46 (56)	
Chemovar monthly dose (ppm)–Terpenoids^#^							
α-Pinene							
Low (3,211–23,413)	149 (35)	58 (39)	29 (30)	19 (27)	19 (25)	25 (30)	12.0 (0.03)
High (23,413–471,725)	125 (29)	44 (29)	33 (34)	31 (44)	33 (43)	33 (40)	
β-Pinene							
Low (2,630–17,807)	145 (34)	57 (38)	25 (26)	23 (32)	25 (32)	27 (33)	5.2 (0.38)
High (17,807–132,146)	129 (30)	45 (30)	37 (38)	27 (38)	37 (35)	31 (38)	
Ledene							
Low (1,071–31,064)	147 (34)	49 (33)	29 (30)	24 (34)	29 (38)	21 (26)	7.0 (0.22)
High (31,064–104,502)	127 (30)	53 (35)	33 (34)	26 (37)	23 (30)	37 (45)	
Limonene							
Low (1,948–55,720)	145 (34)	54 (36)	26 (27)	22 (31)	27 (35)	25 (30)	4.8 (0.44)
High (55,720–414,243)	129 (30)	48 (32)	36 (37)	28 (39)	25 (32)	33 (40)	
Linalool							
Low (1,951–66,760)	137 (32)	52 (35)	29 (30)	26 (37)	29 (38)	26 (32)	1.6 (0.89)
High (66,760–464,197)	137 (32)	50 (33)	33 (34)	24 (34)	23 (30)	32 (39)	
Trans β-Farnesene							
Low (921–25,305)	143 (33)	52 (35)	36 (37)	21 (30)	24 (31)	23 (28)	6.2 (0.28)
High (25,305–84,717)	131 (31)	50 (33)	26 (27)	29 (41)	28 (36)	35 (43)	
α-Fenchol							
Low (1,235–42,024)	150 (35)	51 (34)	23 (23)	21 (30)	28 (36)	26 (32)	8.8 (0.12)
High (42,024–229,057)	124 (29)	51 (34)	39 (40)	29 (41)	24 (31)	32 (39)	
α-Humulene							
Low (5,606–49,291)	141 (33)	52 (35)	27 (28)	23 (32)	30 (39)	27 (33)	3.1 (0.68)
High (49,291–177,085)	133 (31)	50 (33)	35 (36)	27 (38)	22 (29)	31 (38)	
α-Terpineol							
Low (1,342–37,067)	146 (34)	51 (34)	27 (28)	24 (34)	30 (39)	24 (29)	5.1 (0.39)
High (37,067–129,191)	128 (30)	51 (34)	35 (36)	26 (37)	22 (29)	34 (41)	
β-Caryophyllene							
Low (12,327–151,311)	143 (33)	53 (35)	29 (30)	24 (34)	26 (34)	24 (29)	2.7 (0.74)
High (151,311–515,989)	131 (31)	49 (33)	33 (34)	26 (37)	26 (34)	34 (41)	
β-Myrcene							
Low (4,260–75,339)	142 (33)	55 (37)	29 (30)	19 (27)	25 (32)	30 (37)	4.2 (0.51)
High (75,339–577,044)	132 (31)	47 (31)	33 (34)	31 (44)	27 (35)	28 (34)	

IQR, Inter quartile range; mg, mgilligrams; ppm, parts per million; T_1_-T_6_, one to six months follow ups, respectively; #, Terpenoid dose does not add up to 100% due to missing GC/MS/MS tests; all statistic tests were Pearson's Chi-squared.

### Coincidence of Time Variant Parameters

**FIGURE 4:**
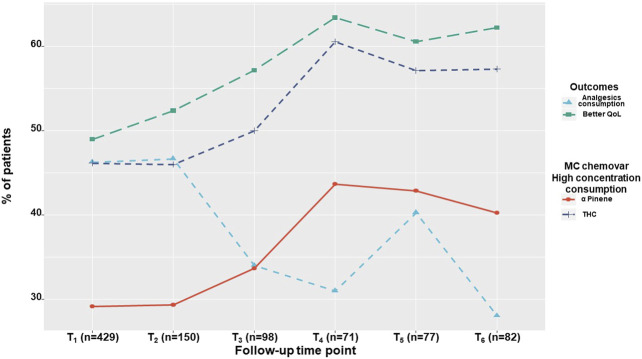
Time-dependent outcome rates. The percentage of patients reporting on analgesics consumption and on 'better' quality of life, alongside with the percentage of patients consuming high THC and *α*-pinene monthly doses, demonstrated for the study sample at one month intervals within a six-month period. QoL, Quality of life; THC, ∆-9-tetrahydrocannabinol; T1, One-month Follow-Up; T2, Two month Follow-Up; T3, Three month Follow-Up; T4, Four month Follow-Up; T5, Five month Follow-Up; T6, Six month Follow-Up.

We found a time dependent increase in better QoL rate and a decrease in analgesic medications consumption rate, and a time-dependent increase in high dose consumption of THC and *α*-pinene rates as shown in Figure 4.

Generalized logistic regression models showed that analgesic medications consumption was associated with THC and *α*-pinene separately. Specifically, patients that consumed a high monthly THC dose, also consumed significantly less (37%) analgesics compared to patients that consumed a low monthly THC dose (46%) (OR 0.18 95% CI = 0.04–0.79; *p* < 0.05). With 429 patients and 905 observations, the model fixed R^2^ was 1% with a mixed R^2^ of 95%. Additionally, patients that consumed a high monthly *α*-pinene dose consumed less (38%) analgesics compared to patients that consumed a low monthly *α*-pinene dose (44%) (OR 0.12 95% CI = 0.02–0.90; *p* < 0.05). With 290 patients and 598 observations, the model fixed R^2^ was 1% with a mixed R^2^ of 97%. However, examined interaction between THC and *α*-pinene in the model was not significant (OR 3.89 95% CI = 0.09–163.33; *p* = 0.47).

**FIGURE 5:**
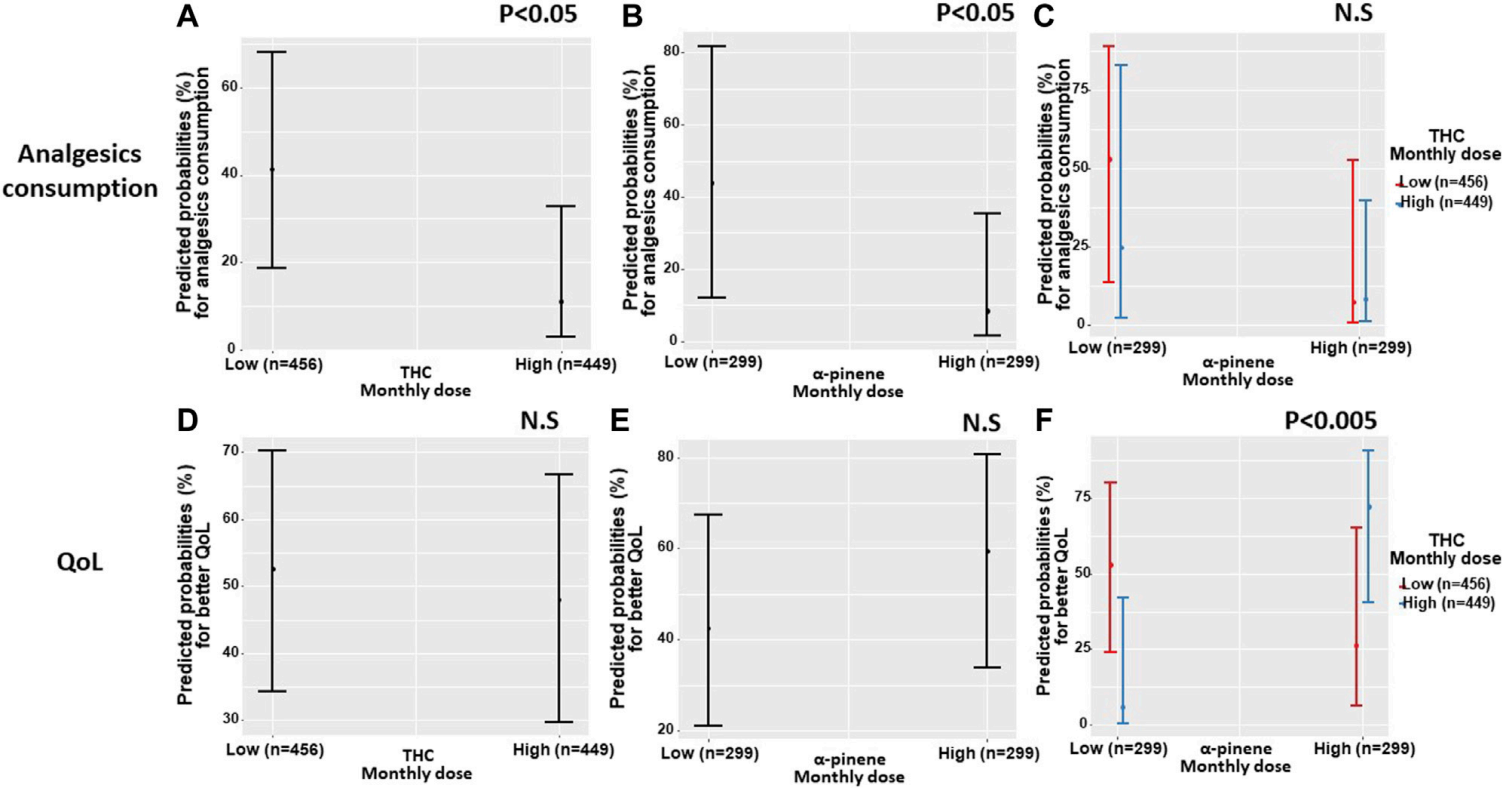
Predicted probabilities of analgesics consumption and quality of life by THC and *α*-pinene dose the predicted probabilities of the associations between the consumption of a high or low monthly doses of THC and *α*-pinene, separately **(A,B)** for analgesics consumption, **(D,E)** for 'better' quality of life) and by interaction **(C)** for analgesics consumption, **(F)** for 'better' quality of life). QoL, quality of life; THC, ∆9-trans-tetrahydrocannabinol; Low THC monthly dose refers to 123–4,892 mg, high THC monthly dose refers to 4,892–14,123 mg; Low *α*-Pinene monthly dose refers to 3,211–23,413 parts per million (PPM), high *α*-Pinene monthly dose refers to 23,413–471,725 PPM.

Generalized logistic regression models showed that THC and *α*-pinene were not associated separately with the observed improvement in QoL (OR 0.83 95% CI = 0.32–2.18; *p* = 0.70 and OR 1.98 95% CI = 0.58–6.79; *p* = 0.27, respectively). However, the interaction between THC and *α*-pinene in the model was significantly associated with the observed improvement in QoL (OR 132.01 95% CI = 4.93–3,531.9; *p* < 0.005), with 290 patients and 597 observations, the model fixed R^2^ was 3% with mixed R^2^ of 92% (Figure 5).

**FIGURE 6:**
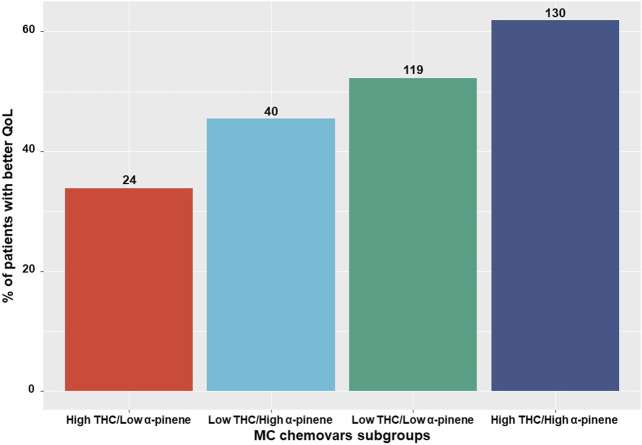
MC chemovar related quality of life reports Subgrouping of the consumed medical cannabis chemovars into low/high THC and *α*-pinene relative to patients' reports on 'better' quality of life. QoL, Quality of life; THC, ∆9-trans-tetrahydrocannabinol; MC, medical cannabis; Numbers on the bars represent the number of patients.

Specifically, patients consuming low *α*-pinene and high THC monthly doses and vice versa (high *α*-pinene and low THC), were less likely to report better QoL scores compared to patients that consumed both high *α*-pinene and high THC monthly doses. Although a moderate positive association exists between THC and *α*-pinene within the unique cultivars that patients consumed in our study (Spearman *r* = 0.35,*p* < 0.05), when evaluating all of the unique cultivar combinations that patients consumed at all time points, a sizable amount of patients (*n* = 64, 15%) consumed contradictory MC cultivar combinations, where THC and *α*-pinene doses were not aligned (i.e., low and high doses for each compound) leading to different rates of QoL between patients (Figure 6). About half of the participants consumed different cultivars at T_6_ compared to T_1._

## Discussion

In this study, we evaluated the reports of CNCP patients under prolonged MC treatment. We observed that while patients' pain intensity reports were stable and did not change throughout this study, their analgesics consumption was reduced and their QoL increased over time. Furthermore, by calculating the patients' monthly dose consumption of specific MC chemovar constituents, we were able to find associations between specific phytocannabinoids and terpenoids' monthly doses consumption and the rates of analgesic medications' consumption and QoL.

In the current study, we found a decrease in analgesic medications consumption and increase in QoL, although pain intensities did not change. This finding could be explained by the cross-sectional study design, in which we had no data on pain intensities prior to the initiation of the MC treatment. These findings are in accordance with Campbell et al. (2018), Sturgeon et al. (2018) that also observed a plateau of pain intensity during cannabis consumption (Campbell et al., 2018, Sturgeon et al., 2018). It is important to note that these authors did not report on the pain intensity of patients prior to initiation of MC treatment. Moreover, contrary to our study, in these studies, participants consumed uncontrolled and illicit cannabis. Nonetheless, ample evidence has been reported on the analgesic effects of MC when information on the pain intensity before MC treatment was reported (Haroutounian et al., 2016; Abuhasira et al., 2018). Thus findings from our study, Campbell et al. (2018), Sturgeon et al. (2018) might not reflect the poor analgesic effect of cannabis, but rather a different perspective of cannabis long-term pain intensity stability.

Although there was no change in pain intensities during the study period, we observed an increase in the QoL of patients. This is in agreement with prospective cohort studies which demonstrated improvement of QoL and sleep quality 6–12 months after initiation of MC treatment (Haroutounian et al., 2016; Zaki et al., 2017). In addition, we observed a decrease in the rate of analgesic medication consumption and a trend in reduction of daily morphine equivalent dose consumption, reflecting previous studies that showed a general decrease in prescribed opioids when MC legislations were approved (Bradford et al., 2018). Our findings strengthen the literature, showing that CNCP patients do not resume conventional pharmaceutical analgesic treatment following prolonged MC treatment.

Participants in this study used 41 unique cannabis cultivars and as they used about two different cultivars a day, a total of 350 different combinations of MC cultivars were possible. According to the participants' monthly dose of MC chemical constitutes (phytocannabinoids and terpenoids) we found an association between THC and *α*-pinene doses and QoL and analgesic consumption. Specifically, the decrease in the rate of analgesic medications was associated with high THC or high *α*-pinene dose consumption separately. The analgesic properties of THC have been extensively reviewed (Aviram and Samuelly Leichtag 2017), while the analgesic effects of *α*-pinene, a bicyclic monoterpene and most widely distributed natural terpenoid (Noma et al., 2010) that is suggested to have anti-inflammatory properties (Liu et al., 2016), have no clinical data to support such properties. On the other hand, the improvement in QoL was associated with consumption of high doses of both THC and *α*-pinene. Despite evidence that essential oils enriched with *α*-pinene can improve mood and cognition (Moss et al., 2010), further research is needed to understand the synergistic effects of THC and *α*-pinene on QoL. Nevertheless, our findings highlight the so called 'entourage effect' of MC (Ben-Shabat et al., 1998). Previous prospective studies have demonstrated QoL improvement and decrease in opioids consumption following cannabis treatment (Haroutounian et al., 2016), however, none of these studies explored which MC compounds may be responsible for these phenomena. Thus, regarding 'cannabis' as if it was a single adherent medication (Rhyne et al., 2016) could lead to major bias due to cannabis treatment complexities with different concentrations of over 90 phytocannabinoids (Baram et al., 2019) and similar amounts of terpenoids (Shapira et al., 2019) between cannabis cultivars (Hazekamp and Fischedick, 2012).

Due to our study design, information regarding dropouts could not be obtained due to ineffectiveness or serious MC-related AEs that might have caused the discontinuation of treatment. Participants reported a long list of non-serious AEs that persisted with their MC treatment. Specifically, 86% reported at least one AE, which is higher than previously reported in long-term cohorts of MC users (Haroutounian et al., 2016; Yassin et al., 2016). Nevertheless, as participants are long-term users of MC, these AEs appear to be tolerated and might not influence the decision to continue using MC.

## Limitations

The current study has several limitations. First, the sizeable dropout rate during our study, and the inability to account for reasons for dropout may represent survival bias. This bias is mitigated by the plateau of most measures during the study period. Additionally, given the prolonged MC treatment duration of the sample, it is likely that the dropout is due to questionnaire fatigue and not due to MC treatment discontinuation. Second, self-report bias may have occurred, although the questionnaire was anonymous and validated, and allowed patients to answer without affecting their current treatment plan. Third, the study design did not allow access to patient data before MC treatment initiation, making it impossible to draw causal conclusions. Fourth, since this study reports only on MC consumption by smoking, the findings could not be generalized to countries where only non-smoking MC products are approved. Finally, as the analyzed MC cultivars were obtained from cultivators and not directly from the individual patients, an estimation bias could be present. Nonetheless, as most cultivars were analyzed several times and we referred only to the largest components and dichotomized them into low/high subgroups, this bias might be diminished.

## Conclusion

CNCP is currently one of the most frequent indications for MC license approval in the world. In this study, although pain intensities did not change under long-term MC treatment, analgesic medication consumption rates decreased and 'better' QoL rates increased. These changes coincided with the increased rates of patients’ consumption of high dose THC and *α* pinene. These results may shed light on the long-term beneficial effects of MC on CNCP. Nevertheless, due to our findings regarding high rates of MC-related AEs, close physician follow-up, even after prolonged treatment durations, is recommended. Future studies should prospectively track patients under MC treatment for a prolonged duration, taking in mind their status prior to MC treatment.

## Data Availability

The raw data supporting the conclusions of this article will be made available by the authors, without undue reservation.
